# The Hospital Microbiome Project: Meeting report for the 2nd Hospital Microbiome Project, Chicago, USA, January 15^th^, 2013

**DOI:** 10.4056/sigs.4187859

**Published:** 2013-07-25

**Authors:** Benjamin D. Shogan, Daniel P. Smith, Aaron I. Packman, Scott T. Kelley, Emily M Landon, Seema Bhangar, Gary J. Vora, Rachael M. Jones, Kevin Keegan, Brent Stephens, Tiffanie Ramos, Benjamin C. Kirkup, Hal Levin, Mariana Rosenthal, Betsy Foxman, Eugene B. Chang, Jeffrey Siegel, Sarah Cobey, Gary An, John C. Alverdy, Paula J. Olsiewski, Mark O. Martin, Rachel Marrs, Mark Hernandez, Scott Christley, Michael Morowitz, Stephen Weber, Jack Gilbert

**Affiliations:** 1 Department of Surgery, University of Chicago Medicine, Chicago, IL, 60637, USA; 2 Argonne National Laboratory, Argonne, IL, 60439, USA; 3 Department of Civil and Environmental Engineering, Northwestern University, Evanston, IL 60208, USA; 4 Department of Biology, San Diego State University, San Diego, CA, 92182, USA; 5 Department of Medicine, Section of Infectious Diseases & Global Health, University of Chicago Medicine, Chicago, IL 60637, USA; 6 Department of Civil and Environmental Engineering, University of California, Berkeley, CA 94720, USA; 7 Center for Bio/Molecular Science and Engineering, Naval Research Laboratory, Washington DC, 20375, USA; 8 Division of Environmental and Occupational Health Sciences, School of Public Health, University of Illinois at Chicago, Chicago, IL, 60612, USA; 9 Department of Civil, Architectural and Environmental Engineering, Illinois Institute of Technology, Chicago, IL 60616, USA; 10 Department of Wound Infections, Walter Reed Army Institute of Research, Silver Spring, MD 20910, USA; 11 Department of Medicine, Uniformed Services University of the Health Sciences, Bethesda, MD 20814, USA; 12 Building Ecology Research Group, Santa Cruz, California, 95060, USA; 13 Department of Epidemiology, University of Michigan School of Public Health, Ann Arbor, MI 48109; 14 Department of Medicine, University of Chicago Medicine, Chicago, IL, 60637, USA; 15 Department of Civil Engineering, University of Toronto, Ontario, Canada; 16 Department of Ecology and Evolution, University of Chicago, Chicago, IL, 60637, USA; 17 Alfred P. Sloan Foundation, New York, NY, 10111, USA; 18 Department of Biology, University of Puget Sound, Tacoma, Washington, 98416, USA; 19 Department of Civil, Environmental and Architectural Engineering, University of Colorado, Boulder, CO, 80309, USA.; 20 University of Pittsburgh School of Medicine, Children's Hospital of Pittsburgh of UPMC, 4401 Penn Avenue, Pittsburgh, PA 15224

This report details the outcome of the 2nd Hospital Microbiome Project workshop held on January 15^th^ at the University of Chicago, USA. This workshop was the final planning meeting prior to the start of the Hospital Microbiome Project, an investigation to measure and characterize the development of a microbial community within a newly built hospital at the University of Chicago. The main goals of this workshop were to bring together experts in various disciplines to discuss the potential hurdles facing the implementation of the project, and to allow brainstorming of potential synergistic project opportunities.

## Introduction

In the United States, nearly 5% of all patients admitted to a medical facility will be diagnosed with a hospital-acquired infection [1]. These infections cost an estimated 36 - 45 billion dollars annually [2] and result in approximately 100,000 deaths annually [3]. While these numbers are concerning, they highlight a considerable lack of evidence regarding both the source and development of nosocomial infections. The Hospital Microbiome Project (HMP) was created to characterize the taxonomic composition of surface-, air-, water-, and human- associated microbial communities at a newly constructed University of Chicago Medical Center Hospital in Chicago, Illinois, USA, also known as the Center for Care and Discovery (CCD) [4]. The aim of the HMP is to sample the building, patients, and staff in a systematic, coordinated approach over the course of one year (January 2013 - January 2014), specifically incorporating building science measurements to determine their influence on the development of microbial communities, with sampling starting one month prior to the opening of the new hospital (February 23^rd^ 2013).

The 1^st^ HMP Workshop was held June 7^th^-8^th^ 2012 to address the initial sampling strategy and approach to building science measurements. This initial workshop made several recommendations and led to the development of a full proposal to the Alfred P. Sloan Foundation as well as to the creation of the Hospital Microbiome Consortium [5]. The final proposal was funded and implemented in December 2012.

Here we present discussion and conclusions from the 2^nd^ HMP Workshop held on January 15^th^, 2013 at the University of Chicago, in Chicago, Illinois, USA. The goals of this workshop were to bring together experts in various disciplines to discuss the current hurdles faced in characterizing interaction between human microbiomes and building surfaces and to consider potential strategies to minimize the transmission of infection diseases within hospitals. The group was comprised of architects, building scientists, and building systems engineers, as well as project managers, medical staff, hospital management, microbiologists, microbial ecologists, virologists, epidemiologists, and standards officials. This represented the last formal organizational meeting prior to the start of sample collection (January 16^th^ 2013). Ample time was also spent brainstorming synergistic project opportunities asking ‘how can the HMP data set complement other studies and how can other studies be incorporated into the HMP?’

The meeting occurred over one day, starting at 9am and ending after dinner at 9pm. The format consisted of two formal presentations with ample time for subsequent discussion; this forum was successful in engaging the research community at the meeting and providing valuable feedback to the project management team. These more formal sessions were complemented by introductions of all meeting participants with an opportunity for each participant to informally describe their interest in the HMP and potential areas for possible synergistic investigations.

**Meeting Participants**
John Alverdy MD: Department of Surgery, University of Chicago, USA
Gary An MD: Department of Surgery, University of Chicago, USA
Seema Bhangar PhD: Department of Civil and Environmental Engineering, University of California Berkeley, USA
Eugene B. Chang MD: Department of Medicine, University of Chicago, USA
Sarah Cobey PhD: Department of Ecology and Evolution, University of Chicago, USA
Betsy Foxman PhD: Department of Epidemiology, University of Michigan School of Public Health, USA
Jack Gilbert PhD: Argonne National Laboratory, USA; Department of Ecology and Evolution, University of Chicago, USA
Mark Hernandez PhD: Department of Environmental Engineering, University of Colorado, USA
Rachael M. Jones PhD: School of Public Health, University of Illinois at Chicago, USA
Kevin Keegan: Argonne National Laboratory, USA
Scott T. Kelley PhD: Department of Biology, San Diego State University, USA
Benjamin Kirkup PhD: Department of Wound Infections, Walter Reed Army Institute of Research, USA
Emily Landon MD: Department of Medicine, Infection Control, University of Chicago, USA
Hal Levin PhD: Building Ecology Research Group, USA
Michael Morowitz MD: Department of Surgery, University of Pittsburgh, USA
Paula Olsiewski: Alfred P Sloan Foundation, USA.
Aaron Packman PhD: Department of Civil and Environmental Engineering Northwestern University, USA
Joan Suchomel: Skidmore, Owings & Merrill LLP, USA
Jeffrey Siegel PhD: Department of Civil Engineering, University of Toronto, Canada
Daniel Smith PhD: Institute for Genomic and Systems Biology, Argonne National Laboratory, USA
Brent Stephens PhD: Department of Civil, Architectural and Environmental Engineering, Illinois Institute of Technology, USA
Mariana Rosenthal MPH, PhD: Department of Epidemiology, University of Michigan School of Public Health, USA
Gary Vora PhD: Center for Bio/Molecular Science and Engineering, Naval Research Laboratory, USA
Stephen Weber MD: Chief Medical Officer, University of Chicago, USA
Ann Womack: Biology and the Built Environment Center, Institute of Ecology and Evolution, University of Oregon, USA

## Session 1: Overview of the Hospital Microbiome Project

### Moderated by Jack Gilbert, PhD

The meeting began with an introduction by project principal investigator Jack Gilbert Ph.D. Jack provided an overview of what he hoped to gain from this meeting. He highlighted the multidisciplinary nature of the project, and applauded the University of Chicago and the management and staff of the new Center for Care and Discovery for helping to pioneer a project, which could have such a revolutionary impact on patient care in hospitals across the globe. He briefly outlined the rationale for the HMP, which is to create a ‘roadmap’ of microbial transmission routes and succession within the hospital infrastructure, to provide researchers with the most detailed exploration of microbial colonization of a new hospital ever under taken. He also thanked the Alfred P Sloan Foundation and his co-convener Capt Benjamin Kirkup for providing the appropriate resources and support needed for the meeting to take place. He thanked his fellow Co-PIs for their support on this award, and the attendees and members of the HMP Consortium for making time to help guide and influence the HMP study design. Finally, he highlighted that while sampling would commence the very next day, everything was still on the table, and having the hospitals Infection and Immunity staff at the meeting would enable vital discussions of technical and political feasibility for any and all suggestions that were raised at the meeting.

## Session 2: Approaches and findings of ongoing microbiome studies

### Moderated by Daniel Smith PhD

Daniel Smith Ph.D. began this session by reinforcing the rationale, presenting existing microbiome data on hospital surfaces and patients, and then discussing the current protocol for the HMP. The existing protocol can be found online at the hospital microbiome website [6] where the full details of the study are provided. Daniel provided an overview of the core hypotheses for the HMP, which were discussed extensively, and found to be appropriate for guiding the research outlined in the existing proposal.

**The core hypotheses were:**
1. *Microbial community structure on hospital surfaces can be predicted by human demographics, physical conditions (e.g. humidity, temperature), and building materials for each location and time.* The *null*-hypothesis states that “microbial community structure exists independently of human interaction and physical conditions of the built system”. While this hypothesis has been tested in different environments, the current study will be testing it to determine if defined relationships exist that could help predict the succession of microbial communities, including potential pathogen reservoirs. Sampling will also be acquired from the health care workers who frequent the patient room to determine if they are vectors in bacterial community formation.
2. *A patient-room microbiota is influenced by the current patient and their duration of occupancy, and shows community succession with the introduction of a new occupant*. The *null-*hypothesis states that “the patient’s interaction with a room has little impact on microbial community structure on the surfaces of that room, and this relationship is not changed by duration of occupancy”. The duration of occupancy for patients in this new hospital will vary considerably. The experimental design will focus on two different modes, oncology (floor 10) will have long residence times of >2-3 weeks, while surgery (floor 9) will have short residence times of <3 days on average. By monitoring 5 patient rooms on these two floors at different temporal scales (daily and weekly) it will be possible to determine the impact of the patient’s occupancy on the composition and structure of the microbial communities in the room.
3. *The colonization of the surfaces and patients by potential pathogens is influenced by composition and diversity of the existing microbial community derived from previous occupants of the space.* The *null*-hypothesis states, “Existing microbial communities associated with a surface do not influence the colonization, succession or development of a new community”. Validation of the hypothesis would indicate that existing microbial communities could be useful in altering the development of new, potentially pathogenic, organisms on a surface. This will be vitally important in determining how microbes interact in this complex, dynamic system, and how we can potentially change the paradigm view of bacteria in a hospital, in that some may be beneficial to a healthy environment.
4. *The rate of microbial succession is driven by demographics, usage and building materials.* The *null*-hypothesis states “community structure and composition evolves independently of human population demographics and occupancy and of the building materials used to construct the room and surfaces”. This hypothesis explicitly focuses on the rate of succession of the community, and will be explored by comparing different temporal resolutions of observation, and defining how rapidly communities change on different surfaces, and in rooms with different patient, and health-care worker turnover rates.

Subsequent discussion began by exploring the potential dynamics governing the development of the microbiome in patient rooms. Initially, the time between occupancy of the patient rooms was discussed with regard to the role this may play in the formation of the room microbiome. Seema Bhangar reviewed work done in classrooms showing that occupancy produced very dynamic changes in levels of airborne biological particles (in a study where laser- induced fluorescence was used as an indicator of biological origin) [7]. She described that when people enter a room, there is an immediate spike in airborne levels of large (1-15 micron) total and fluorescent biological airborne particles. Pronounced changes in the airborne signal are observed during cleaning, and with the change in operational state (off vs. on) of a mechanical ventilation system. Emily Landon, an expert in infectious disease, noted that hospital cleaning regimens are not always identical between rooms, and therefore, which regimen was used needs to be recorded. She also stated that all aspects of the cleaning regime, including chemicals and products used, would be made available to the investigators. Additionally, a ‘timestamp’ of when the cleaning occurred will be provided, as well as the duration of time elapsed between room cleanings. Knowing that surface material has a large influence on the composition of microbial flora, Michael Morowitz suggested that recording building material might also be beneficial in helping describe the formation of microbial communities on surfaces; this was flagged, and the details of the building materials were to be made available for databasing.

The conversation then turned to possible contamination issues. Hal Levin, from the Building Ecology Research Group, described the need to differentiate outdoor from indoor air, even if the outdoor air is filtered. Another concern, expressed by Emily Landon, is the potential contamination by dust residing on the air filter apparatus. Brent Stephens suggested that the use of a particle sampler would be helpful in determining the amount of outside air and dust contamination. Emily also suggested that patient visitors might have a potential impact on the microbial content of patient rooms, although she noted that there is no published evidence of a direct link between hospital attendance and disease acquisition, only circumstantial evidence. John Alverdy suggested that the HMP is in a unique position to be able to prove or disprove the link between hospital attendance and patient disease. It was noted, though, that pinpointing exactly how a patient or hospital worker acquired a bacterial infection might impose a liability. For example, Emily points out that in the Netherlands, healthcare workers are banned from working if they screen positive for methicillin-resistant *Staphylococcus aureus* (MRSA). Although this has reduced the rates of MRSA in the Netherlands, it presents a possible conflict between the HMP and the hospital staff, potentially limiting recruitment [8].

Lastly, there was discussion on sample acquisition. Benjamin Kirkup pointed out that currently the way in which we determine infectious agents (i.e. culture of microbes followed by biochemical virulence testing) ignores the vast majority of the microbial world. The HMP using 16S, 18S, and ITS rDNA amplicon sequencing has the potential to produce valuable insight into defining the *entire* microbial community in each sample, although as noted in the meeting, there are no universal primers [9]. Eugene Chang additionally questioned how the role of viruses would be defined? This highlights a significant problem in most ecological experiments and observations involving microbial communities, which is the lack of understanding of the role viruses play in the dynamics observed for these communities. The problem is that there is no single gene that can be used as a ‘universal’ marker to capture the diversity of phage as they infect and ‘control’ microbial populations. Therefore, the only immediate solution is to either target the analysis and search in the light of current knowledge, or develop techniques to use metagenomic sequencing with enriched viral samples. Jack Gilbert highlighted ongoing work by Scott Kelley to define virus sampling and observation protocols using metagenomics, including data being generated at the current time, and suggested that these will be applied where appropriate to the HMP samples to try and examine how phage are changing bacterial community dynamics in these settings. A singular concern is actually obtaining enough viral particles to obtain enough DNA, as well as how we deal with DNA vs RNA viruses in analysis. Scott Kelley discussed some techniques he is currently working on to address these concerns, however, he also explained that these were not yet published. Emily Landon expressed concern about examining viruses at all, by voicing the common notion that hospitals do a relatively good job disinfecting viruses; however, Gary An questioned ‘how can the hospital do an adequate job of viral disinfection if they do not know what they are looking for?’ Hence, it was agreed that exploring a method for describing viral populations was needed and could potentially be a major NIH proposal to utilize the HMP samples.

Mariana Rosenthal, an epidemiologist from the University of Michigan, alerted the group to the potential biases resulting from choice of sampling methods. For example, hand swabs will likely yield different results from the glove-juice method, whereby the hand is placed in a buffer-containing sterile bag, and massaged for a minute. Swab sample collection efficiencies may depend upon the swab material and swab composition used, the surface type, and how the investigator swabs the surface of interest. It was suggested that videos of the sampling could be made for both training investigators and for exploring variance. Further, it was proposed that during data analysis, a sampling treatment bias analysis be part of the standardization effort. It was finally discussed that while glove-juice, tissue punches and scrapes were absolutely more effective at isolating community information from skin samples, there were certain impracticalities to applying these techniques repeatedly for thousands of samples, and with sampling done every day, the ability of existing personnel to carry out this initiative would be limited. Therefore, for the time being vigorous skin swabbing was upheld as an appropriate strategy. However, the potential to examine the glove-juice method in the context of the HMP was not disregarded, and was in fact encouraged, so that an accessory study was discussed.

## Session 3: Potential litigation issues with hospital research

### Moderated by Stephen Weber MD

Stephen Weber MD, an expert in infectious disease, clinical quality and patient safety, and the Chief Medical Officer of the University of Chicago Medical Center presented his view of the HMP. First, Dr. Weber expressed his great excitement for both the development of HMP and the geographic home for the HMP being the University of Chicago Medical Center. As a hospital administrator, Dr. Weber discussed the potential litigation issues of conducting hospital-based research and performance improvement in Cook County, one of the most litigious areas of the country. He described the Illinois Medical Studies Act (735 ILCS 5/8-2101), a law that defines the appropriate management of data and documents, allowing protection from litigation in support of performance improvement and quality activities. The intent of the law is to encourage clinicians and investigators to engage in free discussion and investigation to improve patient care. Despite this, Dr. Weber stressed the importance of putting in place safeguards when collecting and managing patient data to protect patient confidentiality.

## Session 4: Public perspective on the HMP

### Moderated by Emily Landon MD

Emily Landon led a discussion on the public perspective on the HMP noting the common public notion that microbes are bad, and that we should work towards total disinfection. It was suggested that the HMP has the potential to educate the public, and show microbial ecology in a new light. Yet, due to the potential risk that the public will view this investigation as negative (i.e., microbes are bad and the hospital is dirty), we must be extremely careful in how we disseminate our findings. It was discussed that with the help of infection control groups, ethicists, legal teams, and the HMP consortium, guidelines need to be developed that will help translate the data to the public in a positive light. Overall, the group agreed that an important goal of the HMP should be to provide public education about microbial ecology and how this project may relate to human health and disease.

## Session 5: Meeting participant introductions and brainstorming

The final session was designed to provide an opportunity for open discussion and brainstorming. Each participant was given ample time to describe potential synergistic project opportunities relating to their specific area of expertise. Below is a summary of the main discussion points raised by each participant:

*Eugene Chang MD:* As a physician scientist, Eugene Chang wanted to use the HMP to explore how microbes impact health, and how humans influence microbes. He is especially interested in *Clostridium difficile* infection, a common nosocomial infection of the intestine, and questions if *C. difficile* is present in the gut microbiome of every patient, even if at low abundance, which would allow the pathogen to emerge given a specific microenvironment?

*Gary An MD*: Gary An, a surgeon and computational biologist, was interested in how hospital acquired infections occur, and how ecological forces influence microbial pathogenesis. He discussed the potential of agent based modeling of these forces to gain insight into the dynamics that govern microbial ecology.

*Jeffrey Siegel PhD:* As an architectural and environmental engineer Jeffrey Siegel was interested in looking at the structural parameters of the building itself that influence microbes, and deeply exploring this complex relationship.

*Hal Levin:* As a research architect, Hal Levin suggested that the chemistry on surfaces was vital to understanding the development of microbial communities. Biological interaction with a surface will both be influenced by and will influence this chemistry.

*Michael Morowitz MD*: Michael Morowitz commented that the microbiome is going play a pivotal role as we enter the era of personalized medicine. He has designed a NICU microbiome study and would like to compare his data to this hospital study. These studies allow us to introduce whole genome sequencing to monitor evolutionary development of the microbiome in these various systems.

*Benjamin Kirkup PhD:* Captain Benjamin Kirkup runs the US Army hospital microbiome analysis, which is a sister study of the core HMP. He discussed *Acinetobacter* infection in soldiers, and how, by using swabs of patients, they investigated bacterial spreading patterns [10]. Results demonstrated that the source of *Acinetobacter* was from local nationals, who after interacting with soldiers at field hospitals, infected soldiers. He is now interested in potential routes of transmission in the hospitals, especially hospitals where patients have exceptional, vulnerable wounds. He also suggested the use of auto-fluorescent particle monitoring in which healthcare professionals add the trackable particle to a surface and then monitor its movement around a system.

*Joan Suchomel*: As an architect, she is interested in how building design directly affects patients.

*Scott T. Kelley PhD:* As a biologist, Scott Kelley discussed his interest in the office, NICU, and viral microbiome. His research focuses on investigating the presence of plasmid and free DNA leading to antibiotic resistance.

*John Alverdy MD*: A surgeon scientist, he has identified the molecular pathways by which microbes sense host stress, which in turn activates virulence circuitry increasing pathogenicity. He is very interested in the HMP citing, that there is little evidence as to why patients get infected in hospitals.

S*eema Bhangar PhD*: Deploying real time instruments to measure particles and occupancy, to obtain insight into the role of human activities in the release and spread of particles (including microbes) in a NICU. A central challenge is designing an instrument case that meets the hospital’s constraints.

*Gary Vora PhD:* As a molecular microbiologist and co-developer of the Antimicrobial Resistance Determinant Microarray (ARDM), Gary Vora was interested in testing the HMP sample collection using the ARDM as a surveillance tool to establish the baseline of drug resistance genes in this environment. He felt that when this data was integrated with the many other data types that are to be generated from within the HMP, it would provide a unique opportunity to identify potential reservoirs of antimicrobial resistance and monitor the evolution of drug resistance and multidrug resistant genetic assemblages over time and space.

*Rachael Jones PhD:* Rachael Jones, an expert in exposure science and microbial risk assessment, is interested in using the HMP data to understand how microbes move through the hospital environment, and impact infection risk for health care workers.

*Kevin P. Keegan PhD:* Kevin Keegan, an expert in bioinformatic statistics, was interested in making sure that sampling was robust enough to pick out correlations of interest; even if individual data points/modules (e.g. faulty air sampling in a room) have to be culled. The replicated design that is currently in place addresses these issues. Kevin is also interested in applying and/or developing statistical analyses that will take full advantage of the robust sampling regimen to tease out gross and subtle correlations between various hospital environments and the evolving composition of their microbial communities.

*Mariana Rosenthal MPH, PhD:* Mariana Rosenthal is interested in the hand microbiome of healthcare workers and posits ‘does the hand microbiota mediate pathogen carriage among healthcare workers?’ She suggests that by using real-time qPCR from HMP samples, we will be able to determine the correlation between microbial community structure and the relative abundance of pathogenic taxa.

*Betsy Foxman PhD:* Described the importance of colonization resistance of microbiota and suggested that if the results of the HMP show that the microbiota on surfaces and hands is resilient to cleaning, then we will need to undergo further investigation to determine how and why. To do this, it is imperative to preserve the swab samples to do metagenomics, metatranscriptomics, metametabolomics, and metaproteomics in the future.

*Sarah Cobey PhD:* As an ecologist, Sarah Cobey focuses on mathematical and statistical analyses to infer how pathogens interact with each other and with their hosts. She mentioned several approaches to understanding the flow of microbes within the hospital system. She suggested that accurately characterizing microbial community succession and metapopulation dynamics might require more intensive sampling of healthcare workers. In addition, stool samples could illuminate the transmission of microbes that are potentially transmitted by the fecal-oral route. Although these kinds of intensive samplings are the ‘best case scenario,’ she noted that the protocol should not discomfort or inconvenience the subjects.

*Aaron Packman PhD:* As an environmental engineer, Aaron Packman spoke about pathogen spread in municipal water systems, interaction of pathogen biofilms, biofilm sloughing, and biofilm detachment. He stated that we currently do not understand what impact selective pressures are having on the development of microbial diversity. He would like to do transport modeling to create bacterial network structures of connectivity within the hospital to determine what controls the community that persists over time.

*Mark Hernandez PhD:* As an environmental microbiologist, Mark Hernandez studies microbial particle transport in liquids (i.e. air and water). He would like to sample aerosols in different environments of the hospital, and proposed an extensive analysis within the CCD to compliment and augment results from the core HMP work.

*Brent Stephens PhD*: Presented an overview of the building science measurements that will complement the biological sampling in the hospital for this study. These measurements include active sensing and logging of patient room temperature, relative humidity, and light intensity; patient room pressurization with respect to the adjacent hallway; percentage of outdoor air delivered to each floor; and human occupancy and air exchange rates in each patient room. Additional passive measurements include aerosol sampling of patient room air for microbial analysis using particle filter media covering the return HVAC grilles in each room affixed with strip magnets. The use of high- precision CO_2_ monitors in the central HVAC supply, return, and outdoor air streams provide an estimate of the fraction of outdoor air delivered to each floor and individual flow measurements in patient rooms provide an estimate of outdoor air ventilation rates in each room. The same CO_2_ monitors are used in the patient rooms alongside IR beam break sensors across the doorways to measure patient room occupancy. Active data collection occurs at 5-10 minute intervals throughout the duration of the study. Obtaining these measurements necessitates working closely with building supervisors, physical plant staff, and regulatory bodies in order to install monitoring devices in the patient rooms and the mechanical floors housing the hospital’s HVAC systems.

## Wrap-Up and summary

A number of key projects were identified as described above that could help to augment or support the existing HMP measurements, including viral diversity measurements, increased antibiotic resistance array screening, particle transport measurements, and personnel tagging for demographic movement analysis. However, the existing strategy for the core HMP remained unchallenged, with all participants agreeing that despite certain inherent biases, the resulting data would be highly appropriate for testing the specific hypothesis outlined in the study. All participants were invited for dinner at the end of the meeting, which resulted in lively discussion regarding the potential impact of the study and the importance of continuing this research beyond the existing 2- year study. It was agreed that sources of funding should be identified that could help support and augment the existing research effort. One key research project that was identified revolved around high spatial and temporal resolution observation of microbial and human occupant dynamics. It was agreed that this would enable more specific identification of the routes of transmission between building occupants and building infrastructure. Aaron Packman agreed to help lead this initiative, and with Jack Gilbert and Rachael Jones, has submitted an application to the Chicago Biomedical Consortium. Emily Landon agreed to help address the IRB language and enable access to existing observational data of healthcare worker activity patterns for this effort.
